# Skin Delivery of Kojic Acid-Loaded Nanotechnology-Based Drug Delivery Systems for the Treatment of Skin Aging

**DOI:** 10.1155/2013/271276

**Published:** 2013-12-04

**Authors:** M. L. Gonçalez, M. A. Corrêa, M. Chorilli

**Affiliations:** Department of Drugs and Pharmaceuticals, School of Pharmaceutical Sciences, UNESP, Rodovia Araraquara-Jaú, km 1, Campus, 14801-902 Araraquara, SP, Brazil

## Abstract

The aging process causes a number of changes in the skin, including oxidative stress and dyschromia. The kojic acid (KA) is iron chelator employed in treatment of skin aging, and inhibits tyrosinase, promotes depigmentation. Nanotechnology-based drug delivery systems, such as liquid crystalline systems (LCSs), can modulate drug permeation through the skin and improve the drug activity. This study is aimed at structurally developing and characterizing a kojic acid-loaded LCS, consists of water (W), cetostearyl isononanoate (oil—O) and PPG-5-CETETH-20 (surfactant-S) and evaluating its *in vitro* skin permeation and retention. Three regions of the diagram were selected for characterization: A (35% O, 50% S, 15% W), B (30% O, 50% S, 20% W) and C (20% O, 50% S, 30% W), to which 2% KA was added. The formulations were subjected to polarized light microscopy, which indicated the presence of a hexagonal mesophase. Texture and bioadhesion assay showed that formulation B is suitable for topical application. According to the results from the *in vitro* permeation and retention of KA, the formulations developed can modulate the permeation of KA in the skin. The *in vitro* cytotoxic assays showed that KA-unloaded LCS and KA-loaded LCS didn't present cytotoxicity. PPG-5-CETETH-20-based systems may be a promising platform for KA skin delivery.

## 1. Introduction

Recent studies have highlighted that medical treatment for skin conditions offers benefits for the medical therapy of psychological health because visible skin diseases are correlated with increased rates of depression, anxiety, and low self-esteem in patients [1].

Melasma is characterized by hyperpigmented macules on sun-exposed areas. It is also a skin condition that may cause psychological effects, for example, feelings of shame, anxiety, depression, and social isolation, with a negative impact on social life, emotional wellbeing, physical health, and financial status [1]. Postinflammatory hyperpigmentation, hypermelanosis, and diffuse-acquired and congenital hypermelanosis can lead to the development of melasma [2].

Melanin is a dark pigment produced by the skin cells inside the epidermal layer and is produced by a process referred to as melanogenesis. The first stage in tyrosine oxidation is related to tyrosinase enzyme. When skin is exposed to UV radiation, the formation of melanin pigment becomes abnormal, causing hyperpigmentation, for example, melasma and skin aging, symptoms of which are particularly prevalent in middle-aged and elderly people [3, 4].

Exposure to UV light produces free radicals, releasing proinflammatory cytokines and growth factors, which activate proteases that degrade collagen and elastin [5]. The degradation makes an imperfect repair or invisible “solar scar,” but repetitive exposure to UV light causes the development of a visible “solar scar,” manifesting itself as visible wrinkle traces [6].

Kojic acid (KA) is a well-known antityrosinase agent, which has been efficiently used for skin whitening and widely used to treat hyperpigmentation. In addition, it acts as a chelating agent for ions of transition metals, for example, Cu^2+^ and Fe^3+^. Due to its ability to scavenge free radicals, it is also used for the treatment of wrinkles [3, 4].

Reinitzer was the first to observe an opaque fluid, and later Lehman determined it was a distinct phase of matter that exhibited properties of both liquids and solids, so he proposed the term “liquid crystal” [7]. These structures flow like a liquid but have some order and are frequently characterized like crystalline solids [7].

Liquid crystal systems (LCS) can be classified as lyotropic, when formed by adding solvent, or as thermotropic, when dependent on the temperature [8]. By increasing the concentration of a surfactant, formation of an LCS can occur, although increasing the concentration of the surfactant can form different structures of liquid crystals [9]. These structures or mesophases are known as lamellar, hexagonal, or cubic and are observed by polarized light microscopy [10].

An LCS can present the expected therapeutic response for a prolonged time, improve efficacy, reduce side effects, and interfere with skin hydration [11], Moreover, they are thermodynamically stable and can be stored for long periods without alteration and have a high capacity for solubilizing drugs [11].

This study aimed at structurally developing and characterizing an LCS consisting of water (W) and cetostearyl isononanoate (O) that was stabilized by the surfactant (S) ethoxylated and propoxylated cetyl alcohol (PPG-5-CETETH-20) and contained KA. In addition, this study evaluated this hybrid material's *in vitro* skin permeation and retention to optimize its use in the treatment of hyperpigmentation and skin age.

## 2. Materials and Methods

### 2.1. Materials

The following chemicals were used as received: Kojic acid (Chengdu Wonho, China), cetostearyl isononanoate (MRP, Brazil), and ethoxylated and propoxylated cetyl alcohol, PPG-5-CETETH-20 (Croda, Brazil). Deionized water was prepared by the Millipore Milli-Q plus purification system.

### 2.2. Preparation of Formulations

A phase diagram was constructed by slow titration of 9 preparations (2 g each) blending 10, 20, 30, 40, 50, 60, 70, 80, and 90% of the surfactant (PPG-5-CETETH-20) and 90, 80, 70, 60, 50, 40, 30, 20, and 10% of the oil phase (cetostearyl isononanoate), respectively, with distilled water. The titrated mixture was continuously stirred by hand and heated to 37°C to ensure its homogeneity. During the titration, the systems formed were visually analyzed for their transparency, opacity, viscosity, and phase separation; the different regions were delineated in the phase diagram. The titrations were performed at a controlled temperature (25 ± 0.1°C). After each phase transition, the samples were maintained at 25 ± 0.1°C for 24 h to equilibrate the system. The percentages of the three components were calculated after each new addition of water to define the boundaries between the regions of the ternary phase diagram [12].

Three regions of the diagram were selected for characterization by polarized light microscopy (PLM), texture testing, and bioadhesion. In the selected systems, KA was incorporated at a concentration of 2%.

### 2.3. Physicochemical Characterization

#### 2.3.1. Polarized Light Microscopy

Samples were prepared by placing a drop of the formulation between a cover slip and a glass slide. They were then examined under polarized light. An Optical Jenamed 2 microscope, (Carl Zeiss, Jena, Germany) was used to analyze the various fields of each sample at room temperature. The isotropic behavior of the samples was observed, and pictures were taken at a 20,000x magnification at room temperature.

#### 2.3.2. Assay Texture

This test was performed with a TA.XT *Plus* texture analyzer (Stable Micro Systems, Surrey, England). The formulations were weighed (15 g for all samples) in 50 mL centrifuge tubes (Falcon, BD, Franklin Lakes, USA) and centrifuged at 4000 rpm for 10 min (Sorval TC 6 centrifuge, DuPont, Newtown, USA) to eliminate air bubbles. The sample was placed below the 10 mm analytical probe, which was lowered at a constant speed (1 mm/s) until it reached the sample. Contact was detected by a 2 mN triggering force, and then the probe continued down at a constant speed of 0.5 mm/s to a depth of 10 mm into the sample. Next, the probe returned to the surface (0.5 mm/s), and after 5 s, a second compression begun. The test provides a force-time curve from which mechanical parameters can be calculated, including hardness, compressibility, adhesiveness, and cohesion. Three repetitions of the experiments were analyzed at 32 ± 0.5°C.

#### 2.3.3. Bioadhesion Test

This test was performed with a TA.XT *Plus* texture analyzer (Stable Micro Systems, Surrey, England) by the test “Hold Time Until.” As the experimental model, we used a domestic pig ear skin purchased from a local slaughterhouse.

The ears were cleaned with water (25 ± 0.5°C), and the ears with injuries were discarded. The undamaged skins were removed from the cartilage with a scalpel, and a 500 *μ*m thick layer of stratum corneum and epidermis was separated from the adipose tissue with a dermatome (Nouvag TCM 300, Goldach, USA). The prepared skins were frozen at −20°C and stored no longer than 4 weeks. On the day of the experiment, the skin was thawed in a phosphate buffer solution, pH 7.4 at 25 ± 0.5°C for 30 min; then, its hair was cut with scissors.

The formulations were weighed (7.5 g for all samples) in 50 mL centrifuge tubes (Falcon, BD, Franklin Lakes, USA) and centrifuged at 3500 rpm for 3 min (Sorval TC 6 centrifuge, DuPont, Newtown, USA) to eliminate air bubbles.

The skin was fixed to the probe with the aid of a rubber ring, and the Falcon tube was fixed below the probe. The sample was placed below the 10 mm analytical probe, which was lowered at a constant speed (1 mm/s) until it reached the sample (detected by a 2 mN triggering force) and remained in contact with the formulation for 60 seconds. Then, the probe was removed slowly at a speed of 0.5 mm/s, and the force exerted by the probe on the skin surface, leaving a formulation, results in curve bioadhesive force versus time. Assays were performed in triplicate.

### 2.4. Development of Analytical Methodology for Quantification of KA in Receptor Fluid 

#### 2.4.1. Determination of the Wavelength of Maximum Absorption of KA in the Ultraviolet-Visible (UV-VIS) Spectrum

A solution of KA was prepared in phosphate buffer at a concentration of 100 *μ*g/mL. Then, a solution made from 2.0 mL of phosphate buffer (0.2 M, pH 7.4) and 400 *μ*L of the 100 *μ*g/mL KA solution was loaded into a quartz cell. After homogenization, a spectrophotometric reading in the wavelength range from 200 to 400 nm was performed. Absorption of cetostearyl isononanoate and ethoxylated and propoxylated cetyl alcohol was also observed to verify whether they could interfere in the quantification of KA.

#### 2.4.2. Analytical Curve of KA in Phosphate Buffer and Methanol

To obtain the calibration curve of KA, we prepared a stock solution of 50 mL of a phosphate buffer solution with a concentration of 100 *μ*g KA/mL in a volumetric flask.

In volumetric flasks, 10 mL solutions with different concentrations of KA were prepared in triplicate by adding increasing volumes of stock solution with the aid of an automatic pipette. The volumetric flasks were filled to the meniscus line with phosphate buffer solution. The reading of the solutions was performed in a spectrophotometer at a wavelength of 268 nm (demonstrated in Section 3.3.1).

Thus, it was possible to draw a graph relating the absorbance versus concentration of KA and calculate the equation of the line and the linear regression coefficient. The aforementioned procedure was used except that methanol was the solvent.

### 2.5. *In Vitro *Skin Permeation Test

For this test, we used pig ear skin from a slaughterhouse immediately after slaughter of the animal and an automatic Franz diffusion cell (Microette Plus).

The ears were cleaned with water (25 ± 0.5°C), and the ears with injuries were discarded. The undamaged skins were removed from the cartilage with a scalpel, and a 500 *μ*m thick layer of stratum corneum and epidermis was separated from the adipose tissue with a dermatome (Nouvag TCM 300, Goldach, USA). The prepared skins were frozen at −20°C and were stored no longer than 4 weeks. On the day of the experiment, the skin was thawed in a phosphate buffer solution, pH 7.4 at 25 ± 0.5°C for 30 min; then, its hair was cut with scissors.

The skin was mounted in a Franz diffusion cell with the stratum corneum facing the donor compartment (where the formulation was applied), whereas the dermis faced the receptor compartment. The latter compartment was filled with 0.2 M phosphate buffer at pH 7.4, which can be used as the receptor fluid [13]. The receptor phase was stirred constantly at 200 ± 0.2 rpm and 37 ± 0.5°C.

A portion of 0.33 g of the formulations containing 2% of KA was spread evenly over the entire membrane area. The volume of the medium receptor was 7 mL and the area available for diffusion was 1.77 cm^2^. The stirring system was activated, and samples of 2.0 mL of the receptor phase were collected at 5, 10, 15, 20, 25, 30, 60, 120, 180, 240, 300, 360, 420, 480, 540, 600, 660, and 720 minutes. To maintain “sink condition,” each collection system was reset to 2.0 mL of the receptor solution.

The experiment was conducted in hexaplicate at 32 ± 0.5°C, and the receiver solution was constantly stirred at 300 ± 0.2 rpm. To calculate the amount that permeated (*Q*), we considered the dilutions that occurred after the first sampling, using (1) described below, according to Aronson [14] and Sato [13]:

(1) Qreal,t=Cmeasured,t·Vr+Σn−1·Ca·Va,

where *Q* is the cumulative amount permeated; *Q*_real,*t*_ is the true value at time *t*, *C*_measured,*t*_ is the measured concentration of collecting at time *t*, *V*_*r*_ is the volume of the receiver solution of the diffusion cell, *C*_*a*_ is the concentration the sample removed, and *V*_*a*_ is the volume of the sample removed.

Quantitation of KA in the phosphate buffer was carried out by UV spectrophotometry at a wavelength of 268 nm (Hitachi), using the calibration curve determined in phosphate buffer, demonstrated in Section 3.3.2 to determine the concentration of KA permeate. The skin samples subjected to the permeation study were cleaned and evaluated for retention.

### 2.6. *In Vitro* Skin Retention

The retention studies were performed using the tape stripping method. After the 12 h *in vitro *permeation assay, the skins were cleaned using soft paper. The stratum corneum was removed using fifteen pieces of tape (Scotch 750, 3M), and the remaining KA was evaluated using solvent extraction. The tape pieces were placed in an assay tube containing 5 mL of methanol and stirred for 2 minutes. Subsequently, the tube was immersed in an ultrasonic bath for 30 min. The solvent was filtered using a 0.45 *μ*m membrane to evaluate stratum corneum (SE) retention. The remaining skin was cut into small pieces and placed in a tube containing 5 mL of methanol, and these samples were then subjected to the same procedure (evaluation of epidermis + dermis (E + D) retention).

Based on the analytical curve in methanol demonstrated in Section 3.3.2, the quantification of retention of KA was carried out by UV spectrophotometry at a wavelength of 268 nm (Hitachi).

### 2.7. *In Vitro *Unspecific Cytotoxicity

Mammal cytotoxicity of the formulations was studied *in vitro* using J-774 mouse macrophages as the cellular model. Cells were seeded at a density of 2.5–10.0 × 10^5^ cells/well in 96-well flat bottom microplates (Nunclon) and exposed for 48 h to different doses of the formulations and the control “KA free” (18.6, 10, 5 e 1 *μ*M). After treatment, the compounds were removed, and the cells were washed once with PBS.

The method of 3 [4,5-dimethylthiazol-2-yl]-2,5-diphenyltetrazolium bromide or MTT is a simple, reliable, and reproducible method to measure the metabolic mitochondrial-dependent reduction of the yellow tetrazolium salt to purple formazan crystals, which are insoluble in aqueous solution by viable cells. For that purpose, the cells and MTT (0.4 mg/mL) were incubated in air at 37°C for 3 h. After the incubation period, the supernatant was removed and formazan crystals were dissolved with DMSO (180 *μ*L). The plates were shaken for 10 min, and the optical densities were measured at 560 nm in a multiwall spectrophotometer. Each concentration was assayed three times, and six additional controls (cells in medium) were used in each test. The data are presented as the IC_50_, the compound concentration required to reduce the cells by half.

### 2.8. Statistical Analysis

The data from the physicochemical characterization tests and skin permeation and retention tests were evaluated by one-way ANOVA and the Tukey's post hoc test with a significance level of 0.05%. Analyses were performed using the Graph Pad Prism software, version 5.01, 2007.

## 3. Results and Discussion

### 3.1. Preparation of Formulations

**Figure 1:**
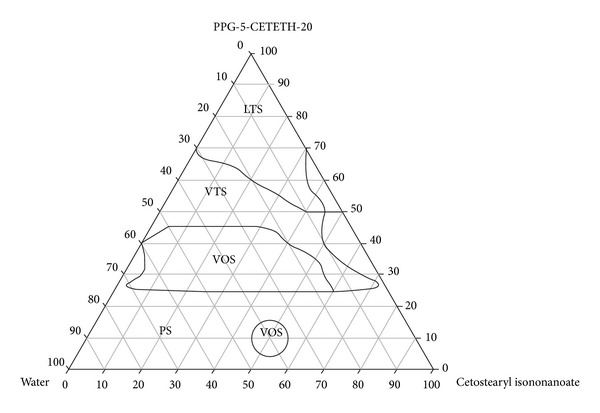
*Phase diagram.* The marked areas represent the following. PS: phase separation; VOS: viscous opaque system; VTS: viscous transparent system; and LTS: liquid transparent system.

For visual characterization of the formulations, the phase diagram illustrated in Figure 1 was created.

### 3.2. Physicochemical Characterization

#### 3.2.1. Polarized Light Microscopy (PLM)

**Figure 2:**
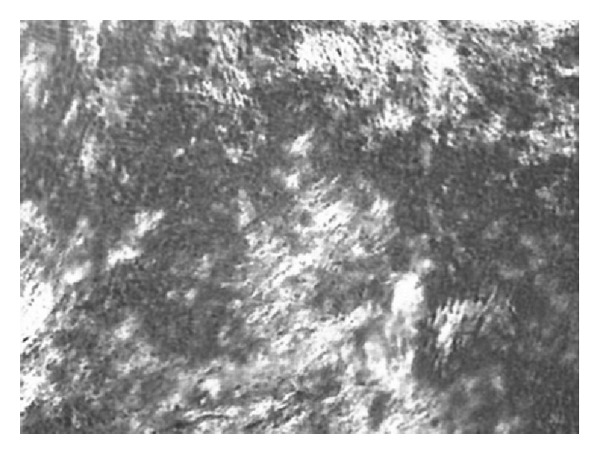
Photomicrograph corresponding to the PLM of LCS lyotropic anisotropic hexagonal mesophases.

**Figure 3:**
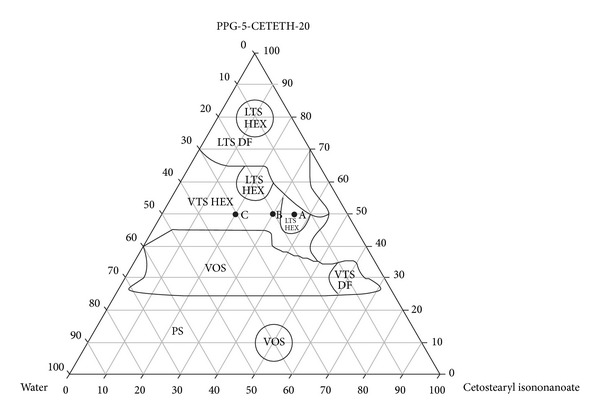
*Systems diagram.* The marked areas represent the following. PS: phase separation; VOS: viscous opaque system; VTS-DF: viscous transparent system dark field; VTS-HEX: viscous transparent system hexagonal mesophase; LTS-DF: liquid transparent system dark field; and LTS-HEX: liquid transparent system hexagonal mesophase.

Structural analysis of the formulations and confirmation that the samples were LCSs were performed by PLM and compared with the characteristics of the hexagonal mesophase, which is illustrated in Figure 2. As a result, the system diagram shown in Figure 3 was constructed.

From the systems diagram, we were able to select the formulations most suitable for the incorporation of KA, for which physicochemical characterization and KA permeation performance would be tested.

In regions of VTSs and LTSs, three points on the diagram were selected, which held the surfactants fixed at 50%: A (35% O, 50% T, 15% W), B (30% O, 50% T, 20% W), and C (20% O, 50% T, 30% W). KA at a concentration of 2% was incorporated into the selected systems. The formulations chosen are indicated in the diagram in Figure 3, where its component concentrations are shown in Table 1. Formulations containing KA had the letter F added to their names (e.g., AF, BF, and CF).

#### 3.2.2. Assay Texture

The mechanical properties of formulations with and without the addition of 2% of KA, such as hardness, compressibility, adhesiveness, and cohesiveness, are presented in Table 2.

These mechanical parameters analyze the stress-strain behavior of the formulations, with the possibility to predict the effects on the stresses of the formulations encountered under physiological conditions [15], becoming an indispensable instrument in the study of drug delivery systems for choosing the most appropriate formulation.

Using ANOVA, the formulations A, AF, B, and BF have properties of hardness, compressibility, and adhesion that are similar statistically (*P* < 0.05), indicating that the incorporation of KA was not significant (*P* < 0.05) in the mechanical properties of these formulations.

According to Table 2, the formulations C and CF showed, statistically higher values of hardness, compressibility, and adhesion (*P* < 0.05). When the probe penetrated samples C and CF, which possessed a thicker consistency (similar in aspect to cooled wax), it showed difficulty in penetrating and leaving these formulations. In this case, the incorporation of KA significantly influenced the compressibility of the formulation, and sample CF showed higher resistance to compressibility.

Regarding cohesion, samples A, AF, C, and CF showed, statistically similar values (*P* < 0.05), and the formulations B and BF showed statistically higher values, showing that the incorporation of the drug significantly increased the cohesion of sample B.

Thus, with respect to texture, formulation B is the most suitable to incorporate the drug because it demonstrated greater cohesiveness, which is an important parameter for topical products; it allows a controllable flow through the vial, facilitating its administration.

#### 3.2.3. Test for Bioadhesion

The values found for the parameter bioadhesive force of the formulations studied are shown in Table 3.

With the addition of water into the formulation, a higher consistency was obtained, and the formulations revealed a greater bioadhesive force, which could improve the ability of the product to fix itself onto the skin of the user.

Thus, formulations A, AF, B, and BF have bioadhesive properties that are statistically similar, indicating that the incorporation of KA was not significant (*P* < 0.05) in the bioadhesive force of these formulations. Formulations C and CF presented significantly higher values with respect to the bioadhesive force (*P* < 0.05), in which case the incorporation of KA significantly influences the bioadhesive property, which decreases with the addition of KA.

### 3.3. Development of an Analytical Methodology for Quantification of KA

#### 3.3.1. Determination of the Wavelength of Maximum Absorption of KA in the Spectrum Ultraviolet-Visible (UV-VIS)

**Figure 4:**
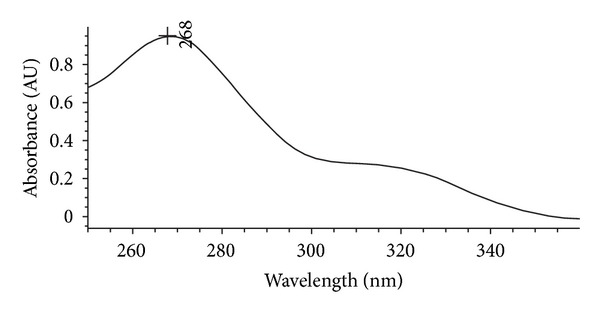
Spectrum scan of KA.

Figure 4 illustrates the spectrum scan of KA, which may observe that the KA has a higher absorption at a wavelength equal to 268 nm.

#### 3.3.2. Analytical Curve of KA in Phosphate Buffer and Methanol

**Figure 5:**
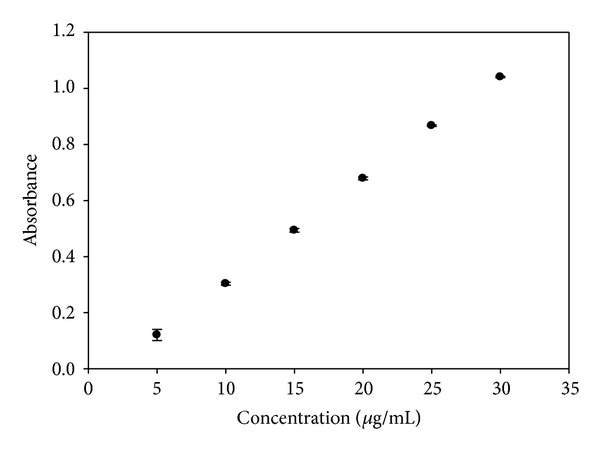
Analytical curve for KA in phosphate buffer.

**Figure 6:**
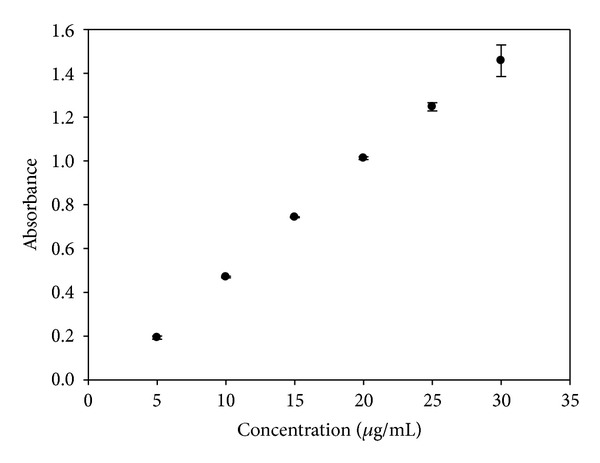
Analytical curve for KA in methanol.

The analytical curve of KA in a phosphate buffer solution was performed to analyze the results of skin permeation because the phosphate buffer solution was used as the receptor solution. The analytical curve in phosphate buffer is illustrated in Figure 5. The analytical curve of KA in methanol was performed to analyze the cutaneous retention of the drug because the KA can be extracted from these tissues with methanol. The analytical curve in methanol is illustrated in Figure 6.

### 3.4. *In Vitro* Skin Permeation and Retention Test

**Figure 7:**
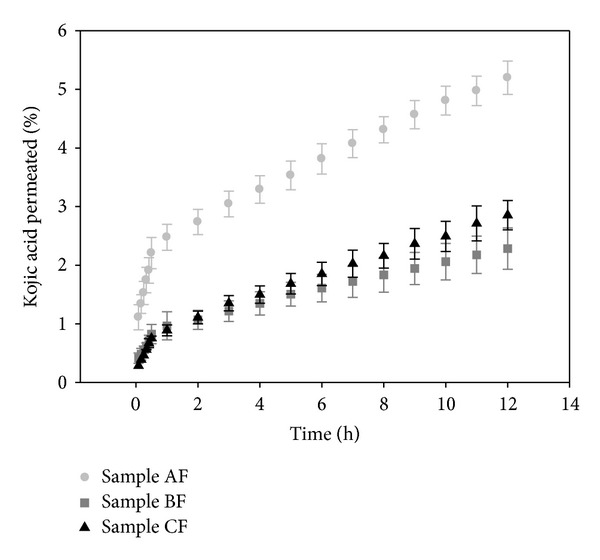
Permeation profile of KA from samples AF, BF, and CF.

Figure 7 presents plots of the release profiles of KA from samples AF, BF, and CF.

Table 4 shows the retention results of KA in SC and E + D.

According to the results from *in vitro* permeation of KA, sample AF presented significantly (*P* < 0.05) greater permeation of KA (5.2%), followed of sample CF (2.8%) and BF (2.3%).

After 12 hours, sample BF retained more drug in the SC (15.4%), followed by sample AF (14.3%) and CF (7.3%), considering that the retention of KA in samples AF and BF is significantly similar (*P* < 0.05) and significantly different from sample CF. As for the E + D, sample AF significantly retained more drug in the stratum (10.2%), followed by sample BF (6%) and CF (2.7%), which are statistically similar values (*P* < 0.05).

This behavior is observed because sample CF has a higher concentration of water, and KA is soluble in water; therefore, KA dissolved in sample CF more than the other. This is shown by the fact that this formulation retained the most drug and showed low permeation and lower retention in SC and E + D.

Unlike sample CF, sample AF has the lowest percentage of water, solubilizing the smallest amount of the drug, making the KA more “free” to go through the layers of skin and permeate it, shown by the fact that the formulation has the highest permeation of KA and a high retention in SE and E + D.

Because formulation BF has an intermediate value of water, this formulation showed an intermediate value of the retention of KA in SE and E + D.

These results demonstrated that the formulations developed can modulate the permeation of KA in the skin, which is precisely the goal of the development of these systems and can allow the KA to stay longer on the skin, where the sites of action are, and not be absorbed. This avoids possible systemic actions of the drug.

Some assumptions can be made about the mechanism of the influence of LCSs on the penetration of KA in the skin. First, the LCSs used were saturated with KA (2%), which may be a factor responsible for the increased penetration of the drug into the skin after topical application. Second, the surfactant or oil molecules can diffuse on the skin surface and act as permeation enhancers of KA because they disrupt the lipid structure of the stratum corneum. This facilitates the diffusion across the barrier that limits the penetration of substances or because of the increase in the solubility of the drug in the skin; that is, it increases the partition coefficient of the drug between the skin and the vehicle [16].

Sato and colleagues used an emulsion for the incorporation of kojic acid with the nonionic wax constituted by cetearyl isononanoate, ceteareth-20, cetearyl alcohol, glyceryl stearate, glycerin, ceteareth-12, and cetyl palmitate, which was suitable for products with an acidic pH, for example, kojic acid. Such networks form the basis of liquid crystals by forming a series of lamellar bilayers of molecules of surfactant around the oil droplets. The amount of kojic acid permeated through the skin was significantly higher (*P* < 0.01) when it was dissolved in phosphate buffer of pH 7.4 than in Emulgade wax; in addition, the phosphate buffer at pH 7.4 showed smaller cutaneous retention, possibly because the solution is not lipophilic and does not interact with the lipids present in the skin [13].

Oyafuso incorporated dexamethasone acetate into an LCS that was composed of water, polyoxyethylene 20 cetyl ether, and polyether functional siloxane and evaluated the retention of the drug in the skin. The tests showed that the dexamethasone acetate was retained in the stratum corneum by these systems. This fact is also promising for topical administration of dexamethasone acetate, promoting the local effect of the drug, which may improve patient compliance to treatment of hyperproliferative skin diseases, for example, psoriasis and cancer, minimizing the side effects of this drug. Moreover, these systems with dexamethasone acetate were also evaluated for their toxicity in different cancer cells, resulting in promising results for cancer treatment [17].

Phelps and colleagues showed that a formulation based on BRIJ and propylene glycol absorbs water and forms an LCS hexagonal phase after 2–4 h in contact with an aqueous medium, providing sustained release for 72 h of naltrexone drug used for the treatment of drug addiction [18].

Nesseem developed an LCS lamellar phase for dermal administration of itraconazole, and this system presented a higher antifungal activity compared to other conventional formulations, presenting itself as a highly effective system for the administration of this drug [19].

### 3.5. *In Vitro *Unspecific Cytotoxicity

The initial *in vitro* test is important in the search for a substance that can be used in the future for preclinical trials. Moreover, *in vitro* assays are able to provide initial parameters for subsequent approaches to evaluate feasibility and therapeutic targets.

**Figure 8:**
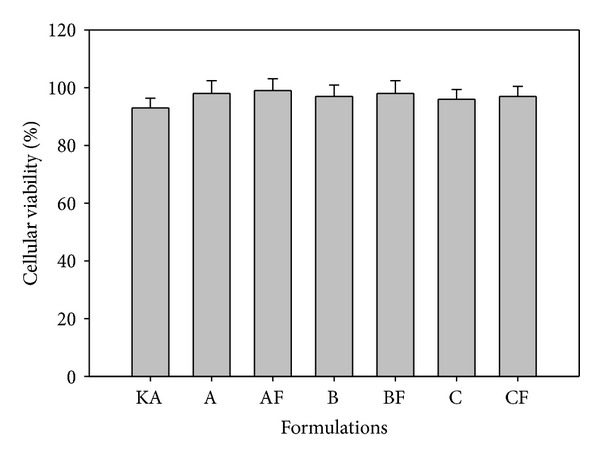
% Cellular viability of free KA, KA-unloaded LCS (A, B, and C), and KA-loaded LCS (AF, BF, and 3F).

*In vitro *cytotoxicity tests were performed for the formulations using J-774 mouse macrophages as the cellular model. The data were exhibited in % of cellular viability, according to Figure 8.

The assay results of % cellular viability showed that the KA free and the formulations were not able to kill normal macrophage cells; all formulations exhibited cellular viability greater than 92%. All of the LCS drugs did not show toxicity, and when the drug was incorporated into the formulations (AF, BF, and CF), cell cytotoxicity was not observed.

## 4. Conclusion

The results revealed that sample CF retained more KA and has a texture profile with a high hardness and cohesiveness, which prevents good product application and produces sensory pleasantness. Sample AF provided good retention of KA for the skin but showed low values of hardness and viscosity, making the administration of the product difficult. Sample BF had a texture profile and bioadhesion suitable for topical use, and its permeation and skin retention demonstrated that this formulation helps modulate the permeation of KA, allowing for its controlled release.

As we have observed, the results suggest that the proposed system is feasible for the incorporation of KA and its controlled release and that the most viable system for the incorporation of KA is system BF. The *in vitro* cytotoxic assays showed that KA-unloaded LCS and KA-loaded LCS do not present cytotoxicity. Therefore, PPG-5-CETETH-20-based systems may be a promising platform for KA skin delivery and to treat topical hyperpigmentation and skin aging.

## Figures and Tables

**Table 1:** Concentrations (in %) of the components of the formulations.

Samples	Oil phase (%)	Surfactant (%)	Aqueous phase (%)
A	35	50	15
B	30	50	20
C	20	50	30

**Table 2:** Mechanical properties of the formulations under study.

Sample	Hardness	Compressibility (kg·s)	Adhesiveness (kg·s)	Cohesiveness
A	8.151 ± 0.768	0.117 ± 0.011	0.040 ± 0.004	0.641 ± 0.0358
AF	8.493 ± 0.477	0.125 ± 0.007	0.038 ± 0.003	0.613 ± 0.0513
B	20.739 ± 1.805	0.287 ± 0.023	0.132 ± 0.016	0.688 ± 0.0339
BF	27.313 ± 1.850	0.392 ± 0.026	0.234 ± 0.020	0.742 ± 0.0188
C	707.643 ± 65.200	9.915 ± 0.859	3.832 ± 1.348	0.614 ± 0.0108
CF	696.091 ± 87.508	10.688 ± 0.878	5.218 ± 2.146	0.620 ± 0.0103

**Table 3:** Values obtained for the parameter bioadhesive force of the formulations under study.

Sample	Bioadhesive force (N ± SD)
A	0.043 ± 0.002
AF	0.034 ± 0.006
B	0.081 ± 0.070
BF	0.047 ± 0.002
C	0.459 ± 0.057
CF	0.209 ± 0.027

**Table 4:** Comparison of retention of KA in the samples for SC and E + D.

Sample	Retention SC [*μ*g/mL ± SD]	Retention E + D [*μ*g/mL ± SD]
AF	14.267 ± 1.487	10.239 ± 1.696
BF	15.365 ± 5.510	5.999 ± 3.197
CF	7.332 ± 3.430	2.698 ± 0.462
